# Effect of fatigue on neuromuscular adaptations in endurance-trained and recreationally active males

**DOI:** 10.7717/peerj.20979

**Published:** 2026-03-17

**Authors:** Hongwei Hao, Rui Wu, Jeremy Liegey, Wen Luo, Jiaping Jiang, Xuanbin Chen, Che Shao, Shijun Liu

**Affiliations:** 1 School of Competitive Sports, Tianjin University of Sport, Tianjin, China; 2 School of Electrical and Electronic Engineering, University College Dublin, Dublin, Ireland; 3 Research Ireland Centre Insight, University College Dublin, Dublin, Ireland; 4 School of Athletic Performance, Shanghai University of Sport, Shanghai, China

**Keywords:** Muscle fibre conduction velocity, Neuromuscular fatigue, Endurance training, Knee extensors, Isometric contraction

## Abstract

**Background:**

Neuromuscular fatigue can be characterized by an exercise-induced reduction in force-generating capacity involving both neural and muscular mechanisms. Previous research has suggested that the functional organization of the neuromuscular system differs between endurance-trained individuals and sedentary or recreationally active individuals. This difference may lead to distinct neuromuscular responses to fatigue. The aim of this study was to compare neuromuscular fatigue responses between endurance-trained (ET) and recreationally active (RA) males during a sustained submaximal isometric knee extension contraction.

**Methods:**

Eleven ET and 11 RA participants performed maximal voluntary isometric contractions (MVIC) of the knee extensors (KE), followed by a trapezoidal contraction (ascending to 60% MVIC) and an isometric fatiguing task at 30% MVIC sustained to task failure. An additional MVIC was completed immediately post-fatigue task. High-density surface electromyography (HDsEMG) was simultaneously recorded from the vastus lateralis, and HDsEMG root mean square (RMS), median frequency (MDF) and muscle fibre conduction velocity (MFCV) were estimated. The MFCV-torque relationship during the ascending phase of the trapezoidal contraction (up to 60% MVIC) was assessed using the mixed linear model.

**Results:**

Baseline MVIC of the KE did not differ between groups. The ET group showed a significantly lower rate of increase in MFCV (*p* < 0.001) during the ascending phase of the contraction and lower absolute MFCV at 60% MVIC (*p* < 0.001) compared to the RA group. During the sustained fatiguing task, both groups reached task failure at similar times with comparable MVIC reductions (∼25%). However, the RA group exhibited significant declines in both MDF (*p* < 0.001) and MFCV (*p* = 0.04), whereas these parameters remained unchanged in the ET group.

**Conclusion:**

While ET and RA individuals exhibited similar levels of fatigue, the underlying neuromuscular mechanisms may differ. The ET group showed a lower rate of increase in MFCV with increasing voluntary force and unchanged MFCV and MDF during fatiguing contractions, whereas the RA group exhibited fatigue-induced decreases in both MFCV and MDF. These findings suggest that endurance training is associated with altered recruitment and/or muscle membrane properties, likely linked to differences in muscle fibre characteristics.

## Introduction

Muscle fatigue is commonly defined as an acute, exercise-induced reduction in the ability to generate maximal voluntary muscle force or power. This complex phenomenon involves a combination of neural and muscular mechanisms, ranging from reduced motor cortical drive to impaired Ca^2+^ handling and slowed cross-bridge kinetics (Enoka & Duchateau, 2016; Gandevia, 2001). Beyond immediate performance impairment, neuromuscular fatigue can compromise neuromuscular control by increasing movement variability and reducing force steadiness (Pethick & Tallent, 2022). These alterations impair proprioception and postural stability, thereby elevating risk of injury (Hadjisavvas et al., 2024). Accordingly, fatigue monitoring and management are increasingly prioritized as central components of performance optimization and injury prevention strategies.

The magnitude and mechanisms of neuromuscular fatigue are highly task-dependent, varying with type, intensity and duration of the task, and spanning limitations in neural drive and impairments in excitation-contraction coupling (Enoka & Duchateau, 2016; Wu et al., 2021). Muscle fatigue and its contributing mechanisms are specific not only to the demands of the task, but also to the physical and physiological characteristics of the individuals assessed, such as the age, sex and training background (Enoka & Duchateau, 2016). For instance, endurance-trained athletes, such as cyclists (Baldwin et al., 1999), distance runners and triathletes (Bontemps et al., 2019), have demonstrated superior fatigue resistance than untrained individuals. Previous comparative studies have indicated that chronic endurance training can alter the relative central and peripheral contributions to fatigue during demanding fatigue tasks (Bachasson et al., 2016). These functional differences are consistent with endurance training-related changes in skeletal muscle, including shifts in myosin heavy chain (MHC) expression and associated fibre phenotypes, as well as the structural and metabolic adaptations that support sustained force production (Plotkin et al., 2021).

To probe these training-related differences mechanistically during voluntary contractions, muscle fibre conduction velocity (MFCV) serves as a valuable non-invasive tool. MFCV represents the average propagation velocity of motor unit action potentials along the sarcolemma of muscle fibres (Casolo et al., 2020b), and is linearly related to the fibre diameter (Blijham et al., 2006). MFCV typically increases linearly with force during ascending voluntary contractions, reflecting the progressive recruitment of larger-diameter, higher-threshold motor units in accordance with the size principle (Andreassen & Arendt-Nielsen, 1987; Del Vecchio et al., 2017), whereas fatigue-related reduction in MFCV commonly suggested impaired membrane excitability (Renaud et al., 2023; Skattebo et al., 2024). Therefore, although MFCV is an indirect measure and reflects the overall propagation behaviour of the active motor unit pool rather than the properties of individuals muscle fibres, it can still provide useful insight into the dynamic changes in motor unit recruitment during fatigue.

Despite the well-documented differences in fibre type composition between ET and untrained populations (Methenitis et al., 2016), the specific behaviour of MFCV during the progression of fatigue remains insufficiently studied. Most existing literature has relied on pre- and post-exercise assessments to infer neuromuscular changes, leaving the recruitment adaptations occurring during the fatiguing task itself largely unexplored. Characterizing these in-task responses is essential for understanding the different strategies that the nervous system employs to maintain power output and for designing training interventions that target specific fatigue mechanisms. To the best of our knowledge, no studies to date have examined the neuromuscular responses during isometric fatiguing tasks in individuals with and without endurance-training background.

The primary aim of this study was to compare fatigue-related neuromuscular responses in endurance-trained (ET) and recreationally active (RA) males during a sustained submaximal isometric knee extension task. A secondary aim was to compare the linear relationship between MFCV and voluntary force during the ascending phase of a trapezoidal contraction. We hypothesized that (1) RA participants would exhibit greater fatigability following the task compared to the ET participants; and (2) ET participants would demonstrate lower absolute MFCVs and a lower rate of MFCV increase with rising force compared with RA participants.

## Materials & Methods

### Participants

Eleven ET distance runners and eleven RA males volunteered to participate in the study. The inclusion criteria for the ET group were: (1) engagement in distance running training for at least 12 months; (2) a minimum of three training sessions per week; and (3) the accumulation of more than 50 km of running per week. The RA group was classified by Participant Classification Framework (Mckay et al., 2022) and was estimated using the Interaction Physical Activity Questionnaire (IPAQ, long form). All RA participants reported at least 150 to 300 min moderate-intensity activity or 75 –150 min of vigorous-intensity activity a week, but they had not engaged in any endurance or strength training in the past 12 months. None of the participants had a history of neurological, cardiovascular, circulatory or orthopaedic disorders. All participants provided written informed consent before testing. The physical characteristics of the participants are presented in Table 1. This study was approved by the Human Research Ethics Committee of Shanghai University of Sport (No. 102772025RT030) and was conducted in accordance with the Declaration of Helsinki (December 2013, Brazil).

**Table 1:** The physical characteristics of the participants (mean ± SD).

	RA (*n* = 11)	ET (*n* = 11)	*p* values
Age (yr)	23.0 ± 1.7	20.1 ± 2.3	0.008
Height (cm)	179.4 ± 4.4	176.9 ± 4.5	0.186
Body mass (kg)	75.4 ± 8.8	64.9 ± 4.8	0.002
BMI (kg m^−2^)	23.4 ± 2.5	20.7 ± 1.4	0.006
Years of training	–	3.0 ± 1.7	
Sessions per week	–	5.4 ± 1.7	

### Mechanical recording

Maximal and submaximal fatiguing contractions of the knee extensors in the right leg were assessed at 90° of knee flexion with the hip fixed at 100° (0° = full extension) using a knee dynamometer equipped with a load cell (AF1; GICAM, Gravedona ed Uniti, Como, Italy, range 0–250 kg, output 2 mV/V). Participants were seated in a rigid chair with their pelvis and trunk firmly strapped, and their arms crossed over their chest. Their lower leg was secured to the dynamometer’s lever arm approximately two cm above the lateral malleolus. For the submaximal fatiguing task, participants maintained a force corresponding to 30% MVIC, matching a horizontal target line (±5% tolerance) displayed on a monitor placed 1.5 m in front of them, with constant visual gain. The task terminated when force dropped >5% below the target for more than 2 s, despite verbal encouragement. Contraction duration was recorded as time-to-task failure (TTF). Force signals were amplified (×100) and sampled at 2,048 Hz *via* a single-channel amplifier (Forza; OT Bioelettronica, Torino, Italy).

### High-density surface electromyography recording

High-density surface electromyography (HDsEMG) was recorded from the vastus lateralis (VL) muscle with a two-dimensional grid of 64 electrodes (5 columns × 13 rows; interelectrode distance of eight mm; GR08MM1305, OT Bioelettronica, Torino, Italy). The grid orientation and position were first adjusted as previously described (Casolo et al., 2020a) using a 16-electrode dry array (silver bars; one mm thick with an interelectrode distance of five mm; OT Bioelettronica) to locate the innervation zone in the distal VL muscle. The anatomical direction of the muscle fibres was then visually inspected by an experienced investigator. After shaving, light abrasion and cleaning with ethyl alcohol, the grid was attached between the proximal and distal tendon regions of the VL muscle, aligned longitudinally with the muscle fibre orientation as previously described (Valli et al., 2025). HDsEMG signals were recorded in monopolar mode and digitised using a 16-bit multichannel amplifier (EMG-Quattrocento; OT Bioelettronica, Torino, Italy; 3 dB bandwidth 10–500 Hz) and sampled at 2,048 Hz. Force and HDsEMG were synchronized, and all data were stored offline for further analysis. Force and HDsEMG signals processing was performed using custom-written MATLAB scripts (R2023b; MathWorks, Natick, MA, USA).

### Experimental design

#### Familiarization

Participants attended a single test session at the research facility. Participants first completed a warm-up on a cycle ergometer at 70–80 rpm with low resistance (50–100 W) for 5 min. Participants then familiarised themselves with the study procedures and performed several maximal and submaximal maximal voluntary isometric contractions (MVICs) of the knee extensors (KE). After a 5-min resting, the electrode grid was positioned on the VL muscle, and the participants performed contractions at 30% (×2), 50% (×2), 70 (×2) and 90% (×1) of their perceived effort to further familiarise themselves with the protocol (Nuccio et al., 2020).

#### Pre-fatigue MVIC

Following a 3-min rest, participants performed at least two knee extension MVICs to establish a baseline for maximal torque. Additional MVIC trials were required if the difference between their two best attempts was greater than 5%. These contractions were performed with a 1-min rest period between each attempt. During each attempt, participants were asked to generate force ‘as hard and as fast as possible’ over a period of approximately 3–4 s.

#### Trapezoidal contraction

After the MVIC test, participants performed one trapezoidal contraction up to 60% MVIC. This contraction consisted of an ascending and a descending phase with a linear increase/decrease in force at 5% MVIC/s, and of a 10-seconds plateau at 60% MVIC between the two phases.

#### Fatigue task

Following a 3–5 min rest, or until their RPE fell below 2, participants performed a sustained isometric fatiguing contraction with the knee extensor muscles maintained at 30% MVIC until task failure. Participants were instructed to match a target line as precisely and as long as possible. The fatiguing contraction was terminated when force fell below the target level for a 3-s period.

#### Post-fatigue MVIC

To assess the effect of muscle fatigue, a post-fatigue MVIC was performed immediately after task failure.

#### Rate of perceived exertion

An index of perceived effort, participants’ rate of perceived exertion (RPE) was assessed with the modified Borg 10-point scale (Borg, 1982). The RPE was recorded before and immediately after the isometric fatiguing contraction.

### Data analysis

#### Force signal

The force signal was firstly converted into torque values (Nm), and low pass filtered using a 4th order Butterworth filter with a cutoff frequency of 30 Hz. The offset was removed by gravity correction. For the MVIC task, the maximal torque values were calculated as the highest 500 ms average reached within each torque recording. To assess the effect of muscle fatigue, post-fatigue MVIC values were normalized to pre-fatigue MVIC. For the trapezoidal contraction, the torque values were normalized to pre-fatigue MVIC in order to gain %MVIC for further analysis.

#### HDsEMG signals

HDsEMG signals were band-pass filtered between 20 and 500 Hz using a 4th Butterworth filter and then converted to single differential signals along the fibre direction (*i.e.,* along the columns of the electrode grid).

**MVIC task**: Electromyography (EMG) root mean square (RMS) and median frequency (MDF) were calculated over a 500-ms epoch corresponding to the highest obtained MVIC and then averaged across the 59 bipolar channels.

**Sustained fatiguing contraction task:** EMG RMS, MDF and muscle fibre conduction velocity (MFCV) were estimated over a 500-ms moving window with 50% overlap from the onset of the contraction until task failure. RMS, MDF and MFCV values were extracted at time points corresponding to 10% intervals of the total fatiguing contraction duration for statistical analysis.

**Figure 1:**
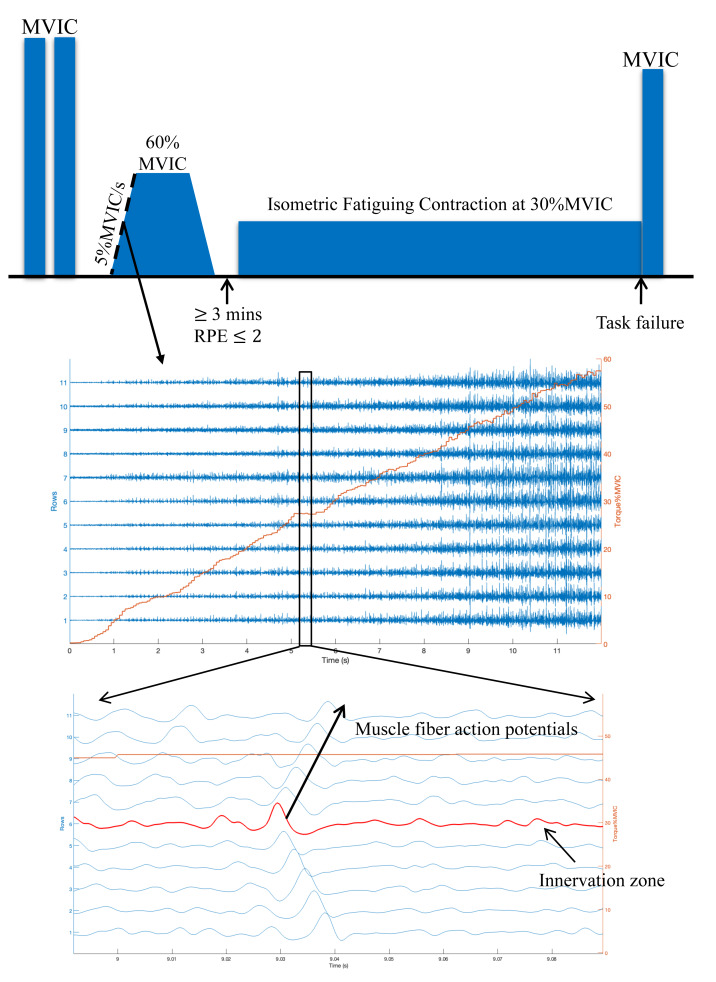
Experimental design overview. (A) The order of the experimental tasks performed by each subject. (B) Representative data from one distance runner during the ascending phase of the trapezoidal contraction at 60% MVIC. Double differential surface EMG signals (blue lines) from the vastus lateralis muscle can clearly be observed as a function of the voluntary force (red line) increases. (C) The innervation zone and muscle fibre action potential propagating in the distal direction.

**Trapezoidal contraction task:** MFCV was additionally estimated during the ascending phase of the trapezoidal contraction up to 60% MVIC (Fig. 1) to characterise the MFCV-torque relationship.

For MFCV estimation, monopolar HDsEMG signals were band-pass filtered between 20 and 500 Hz using a 2nd order Butterworth filter, then converted to double-differential signals, and processed using a multichannel maximum-likelihood algorithm (Farina et al., 2004) with a 500-ms window and 50% overlap. The 4–6 channels with the highest coefficient of correlation (CC ≥ 0.8) were manually selected for MFCV computation (Del Vecchio, Bazzucchi & Felici, 2018a). Estimated MFCV values exceeding the physiological range (2–6 m/s) were excluded from further analysis (Nuccio et al., 2020).

### Statistical analysis

Statistical analyses were performed using R (RStudio v2025.05.0; RStudio Team, 2025). The Shapiro–Wilk test was used to assess normality, and Levene’s test to test for homogeneity of variance. Variables that violated normality assumptions (age, height, TTF and pre-fatigue RMS) were compared between groups (RA and ET) using the Mann–Whitney *U* test, whereas normally distrusted variables were analysed using independent-samples t-tests. To examine fatigue-related changes in MVIC, EMG RMS and MDF, repeated-measures analysis of variance (ANOVA) was used with fatigue (pre- *vs.* post-fatigue) as the within-subject factor and group (RA *vs* ET) as the between-subject factor. Linear mixed models (LMMs) were used to account for the repeated-measures structure of the data and inter-individual variability. The MFCV-torque relationship during the ascending phase of the trapezoidal contraction up to 60% MVIC was examined using group, torque (%MVIC) and their interaction as fixed effects, with participant-specific random intercepts and slopes (MFCV ∼group ×%MVIC + (%MVIC —participant)). To assess group differences in EMG RMS, MDF and MFCV during the sustained fatiguing contraction, LLMs included group, time (continuous) and their interaction as fixed effects, with random intercepts and slopes for time within each participant (var ∼ group × time + (time — participant)). All mixed models were fitted using the *lme4* (Bates et al., 2014) and *lmerTest* (Kuznetsova, Brockhoff & Christensen, 2017) packages. When significant interactions were observed, *post hoc* comparisons with Bonferroni adjustment were performed to control for multiple testing. Effect size was quantified using Cohen’s *f*^2^ with values of 0.02, 0.15 and 0.35 interpreted as small, medium and large effects, respectively (Cohen, 1988). Data are presented as mean ± standard deviation (SD). For graphical presentation, EMG RMS, MDF were normalized to the initial values of the fatiguing task (0%TTF) Statistical significance was set at *p* < 0.05.

## Results

### Baseline measures

At pre-fatigue, the RA and ET participants were similar in height; however, RA participants were heavier with a higher BMI (Table 1). Moreover, knee extension did not differ significantly between two groups (RA: 368.8 ± 87.1 Nm and ET: 294.3 ± 92.1 Nm; *p* = 0.065). Similarly, baseline EMG RMS (RA: 0.142 ± 0.071 mV and ET: 0.198 ± 0.147 mV) and MDF (RA: 76.5 ± 12.4 and ET 70.6 ± 8.6 Hz) were comparable between groups (all *p* > 0.05).

### MFCV-torque relationship

**Figure 2:**
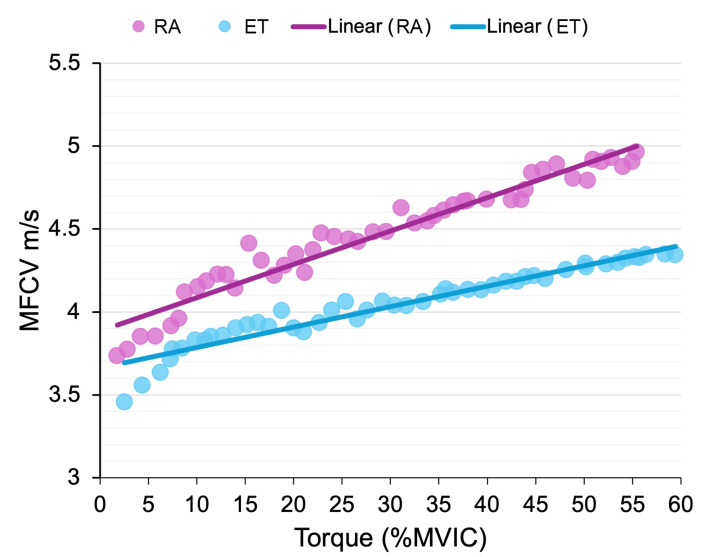
Mean muscle fibre conduction velocity (MFCV) for each group plotted as a function of torque (%MVIC) during the ascending phase of the trapezoidal contraction. The increase in RA (*p* < 0.001) was more pronounced than the increase in ET (*p* < 0.001). Data were presented as the mean for each group at each %MVIC point.

During the ascending phase of the trapezoidal contraction, a significant group × torque interaction was observed in MFCV (*p* = 0.047, *f*^2^ = 0.20). While MFCV increased as a function of relative torque (%MVIC) in both groups, a group by torque interaction was observed (*p* = 0.047, *f*^2^ = 0.20), showing that MFCV increased more rapidly with torque in RA (slope = 0.019 m s^−1^⋅ %MVIC; *p* < 0.001) participants than in ET (slope = 0.012 m s^−1^⋅ %MVIC; *p* < 0.001) participants (Fig. 2). Furthermore, the RA group also showed a higher MFCV at 60% MVIC (plateau phase) than the ET group (4.9 ± 0.3 *vs.* 4.3 ± 0.2 m/s; *p* < 0.001, *η*^2^ = 0.58).

### Effect of fatigue on MVIC

**Figure 3:**
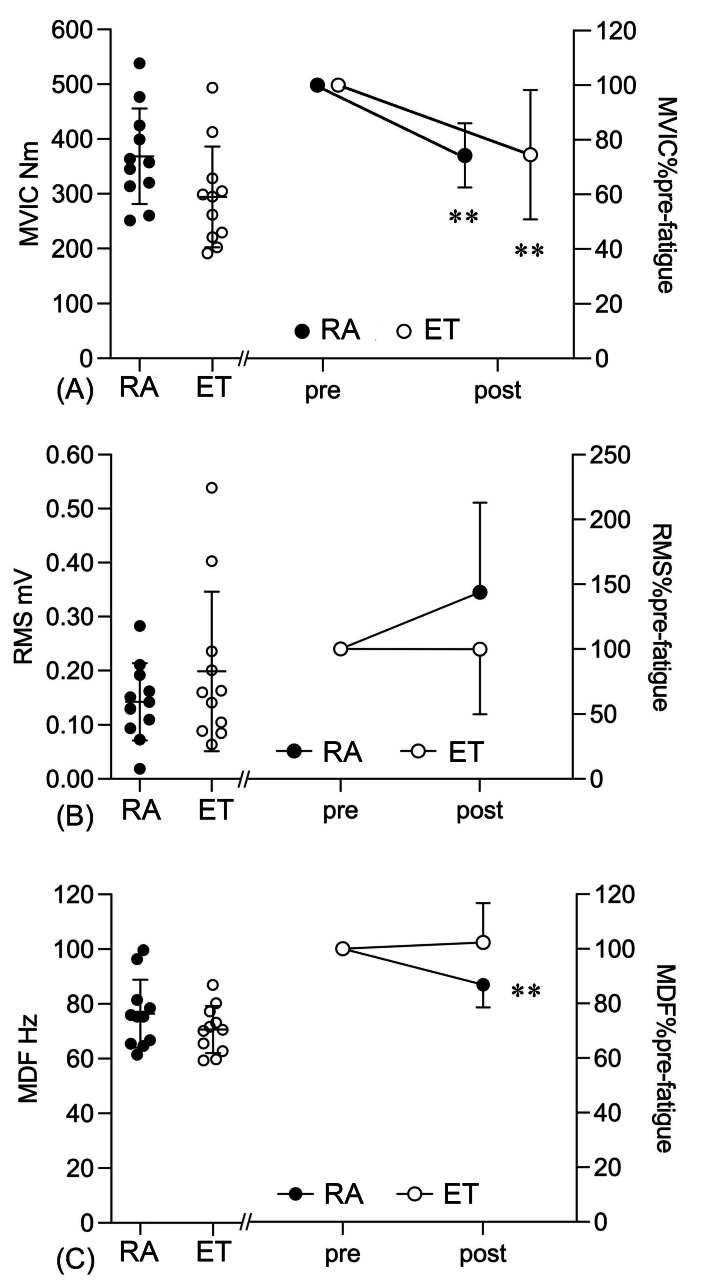
Fatigue-induced changes in MVIC (A), EMG RMS (B) and MDF (C) during MVIC tasks. Note: **p < 0.01 for pre-fatigue compared to post-fatigue.

Following the fatiguing task, RPE increased markedly in both groups (main effect of fatigue: *p* < 0.001; *η*^2^ = 0.98) with similar post-fatigue RPE values in ET (0.9 ± 0.3 to 8.0 ± 1.1) and RA (1.0 ± 0.6 to 8.3 ± 1.0) participants. Similarly, MVIC decreased by approximately 25% in both groups (main effect of fatigue: *p* < 0.001; *η*^2^ = 0.6) (Fig. 3A). In contract, neuromuscular responses differed between groups. A significate group × fatigue interaction was observed for EMG MDF (*p* = 0.006; *η*^2^ = 0.32), showing a post-fatigue decrease in MDF in the RA group (*p* = 0.001), whereas MDF remained unchanged in the ET group (Fig. 3C). No significant changes were observed in post-fatigue EMG RMS in either group (Fig. 3B).

### Fatiguing task dynamics

**Figure 4:**
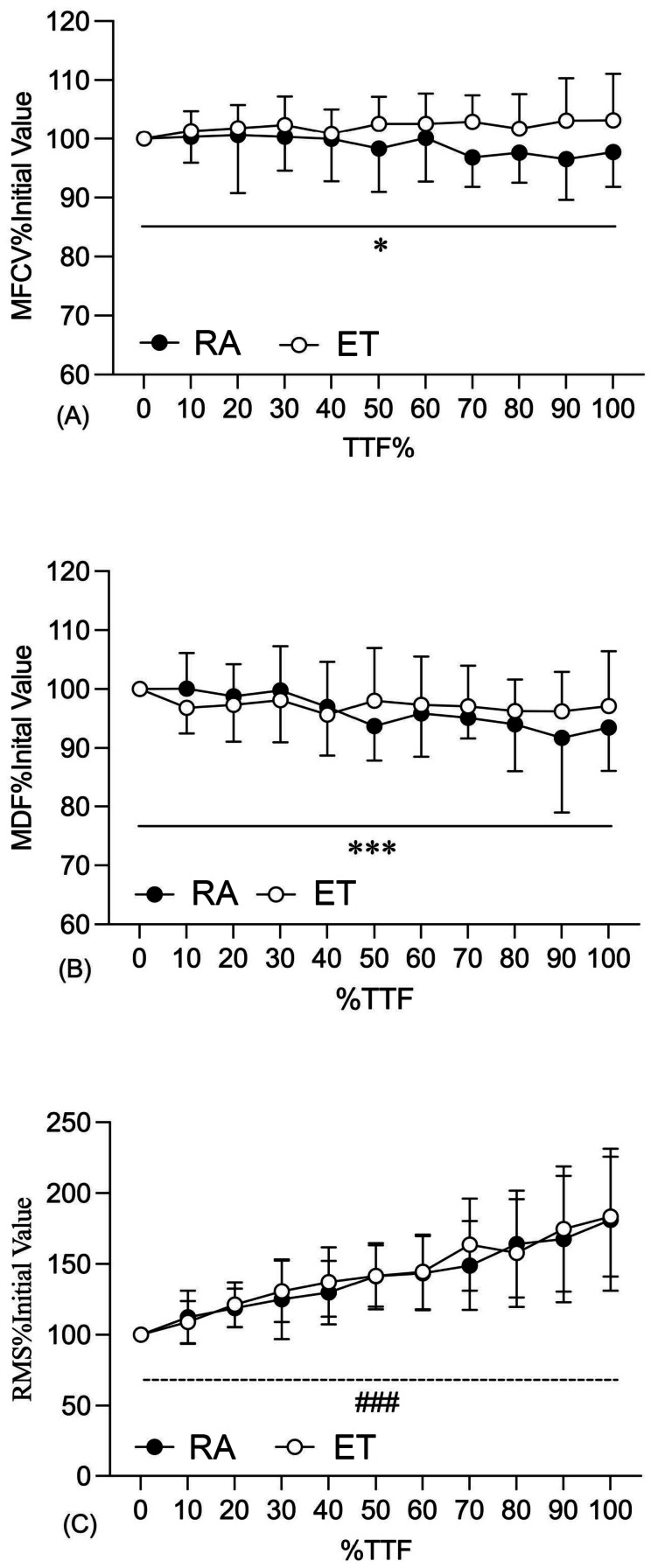
MFCV (A), MDF (B) and RMS (C) during the sustained isometric fatiguing contraction in RA and ET. Note: *p < 0.05, ***p < 0.001 significant decline in RA (time by group interaction); ###*p* < 0.001 significant increase in both RA and ET (main effect of time). Data were presented as the mean and SD for each group.

TTF did not differ significantly between groups (RA: 106.6 ± 71.7 s and ET: 153.3 ± 128.5 s; *p* = 0.511) groups. During the sustained contraction, EMG RMS increased over time in both groups (main effect of time: *p* < 0.001, *f*^2^ = 2.81) (Fig. 4C). However, significant group × fatigue interactions were observed for both MFCV (*p* = 0.032, *f*^2^ = 0.25) and EMG MDF (*p* = 0.012, *f*^2^ = 0.34). Specifically, the RA participants exhibited progressive reductions in MFCV (*p* = 0.047) and MDF (*p* < 0.001) throughout the fatiguing task, whereas these parameters remained unchanged in the ET group (MFCV: *p* = 0.276 and MDF: *p* = 0.504) (Figs. 4A, 4B).

## Discussion

This study investigated the neuromuscular responses to a fatiguing isometric knee extension task in ET runners and RA males. Additionally, we examined the MFCV-torque relationship during linearly increasing isometric voluntary contractions up to 60% MVIC. While RA and ET participants experienced similar overall muscle fatigue, the ET participants exhibited distinct neuromuscular adaptations. In the ET individuals, MFCV and EMG MDF remained unchanged during the fatiguing task and increased less with rising torque. It supports the hypothesis that ET participants develop neuromuscular fatigue more slowly than RA participants, due to a distinct neuromuscular response to fatigue.

### Pre-fatigue (baseline) measures

Interestingly, the knee extension MVIC did not differ between the RA and ET groups at baseline, which was associated with similar levels of EMG RMS and MDF values. Several previous studies have observed comparable maximal knee extension strength between endurance-trained and untrained individuals (Pääsuke, Ereline & Gapeyeva, 1999; Bontemps et al., 2019), which may reflect the nature of the endurance training modality. Endurance training, such as distance running, consists of low-resistance, long-duration aerobic exercise with a high overall volume (Ellefsen & Baar, 2018). This predominantly involves the recruitment of slow-twitch muscle fibres with low force-generating capacity (Costill et al., 1976). Consequently, it leads to physiological adaptations in energy production through oxidative metabolism with no observable changes in muscle or fibre cross-sectional area (CSA), or muscle strength (Farup et al., 2012). Indeed, prolonged endurance training may even interfere with the development of maximal muscle strength (Schumann et al., 2022; Petré et al., 2021).

### MFCV-torque relationship

The MFCV is primarily determined by the size and type of muscle fibres, because larger fibres have lower cytoplasmic resistance, resulting in faster action potential propagation (Blijham et al., 2006). In the present study, the ET group exhibited a lower MFCV at 60% MVIC than the RA group. This difference is likely due to the training-related differences in muscle fibre type distribution and muscle membrane properties. At the muscular level, the conduction velocity of actional potentials is linearly related to muscle fibres diameter (Blijham et al., 2006; Hakansson, 1956). For instance, Sadoyama et al. (1988) have shown a correlation between MFCV and the percentage CSA (%CSA) of type II muscle fibres in the VL (Sadoyama et al., 1988). Indeed, distance runners typically demonstrate a greater proportion of oxidative, smaller diameter fibres. Methenitis et al. (2016) examined ten young sedentary males and nine endurance runners, reporting that the runners had a higher %CSA of type I fibres and a lower %CSA of type II and IIx fibres in comparison with sedentary individuals. These findings support our hypothesis and suggest that ET runners may recruit a greater proportion of type I fibres at the 60% MVIC than their RA counterparts, thereby resulting in a lower MFCV.

We also examined the MFCV-torque relation during the ascending phase of the trapezoid contraction up to 60% MVIC. In both groups, MFCV increased linearly with isometric voluntary torque, however, the rate of increase was less pronounced in the ET compared to RA group (Fig. 2). This linear relationship between MFCV and force has been widely reported in the muscles such as the tibialis anterior (Del Vecchio et al., 2017; Del Vecchio, Bazzucchi & Felici, 2018a), the VL and the vastus medialis (Nuccio et al., 2020) muscles. However, this relationship is, for the first time, compared in individuals with an endurance training background and those without. Del Vecchio et al. (2017) indicated that the linear increase in MFCV with voluntary force is related to the progressive recruitment of motor units with higher conduction velocities, due to the size-dependent, progressive orderly recruitment of motor units (Del Vecchio et al., 2018b). Therefore, our results suggest that, as voluntary force increases, ET males may preferentially recruit motor units with lower thresholds and smaller diameters as force increases, in line with their greater proportion of type I fibres.

### Effect of fatigue between RA and ET groups

Participants in both the ET and RA groups exhibited a comparable level of fatigability, characterized by similar fatigue-induced declines in MVIC (∼25%) and TTF. This conflicts with our original hypothesis and with the results of previous studies that have compared endurance-trained and untrained individuals (Pääsuke, Ereline & Gapeyeva, 1999; Bontemps et al., 2019). For instance, Pääsuke, Ereline & Gapeyeva (1999) found that endurance-trained athletes had a longer TTF than in untrained and power-trained males during submaximal sustained isometric contractions (Pääsuke, Ereline & Gapeyeva, 1999). Similarly, Bontemps and colleagues (2019) found that endurance-trained athletes completed more repetitions than untrained males during intermittent maximal voluntary tasks. One possible explanation for this discrepancy is differences in training background and exercise across studies. In the study by Pääsuke, Ereline & Gapeyeva (1999), the athletes had accumulated 7–15 years of endurance traning, and in the study by Bontemps et al. (2019) the endurance-trained group comprised international level athletes who trained at least six times per week. In contrast, the distance runners in the present study had an average of approximately three years training experience and trained approximately five sessions per week, which is more representative of a recreational rather than elite athlete profile.

Despite similar post-fatigue torque reduciton, the underlying neuromsuclar strategies adopted to maintain submaxmial torque differ between groups. The RA group demonstrated fatiuge-related declines in MDF and MFCV during the sustained fatiuging contarction (Figs. 4A, 4B) and a lower MDF during the post-fatiuge MVIC (Fig. 3C), whereas these parameters remained stable in the ET group. It suggests that, compared with RA participants, ET participants may have developed less preipheral fatigue and recrutied less additonal motor units to sustain the target torque. A similar study has been reported by Callewaert and colleagues (2013), where untrained controls showed a greater decline in mean power freqeuncy (MPF) during repeated submaixmal (30%–40% MVIC) knee extension contracitons, while trained youth sailors exhibited a more stable MPF response. Endurance training has been reported to improve the aerobic capacity of localized muscle groups by enhancing metabolite clearance (Brodal, Ingjer & Hermansen, 1977) and shifting muscle fibre distribution towards a higher proportion of oxidative type I fibres (Howald et al., 1985). Such adaptations could result in a higher strength endurance capacity but not in a higher maximal strength capacity (Petré et al., 2021), which is consistent with our finding. Furthermore, the concurrent reductions in MFCV and MDF observed in the RA group likely refect fatigue-related impairment in membrane excitability. Sustained isometric fatiguing contarction can disrupt ionic homeostasis (*e.g.*, accumulation of extracellular K^+^ and intracellular Na^+^) and increase metabolite-related acidosis, thereby reducing sarcolemmal excitability (Renaud et al., 2023) and slowing action potential progagaion, and shifting the EMG spectrum toward lower frequencies (Rampichini et al., 2020). In contrast, the maintained MFCV and MDF in the ET group may be partly explained by training-induced upregulations of Na^+^/K^+^-ATPase pump activity and improved metabolic buffering capacity (Skattebo et al., 2024). These adaptations would enhance the maintenance of ionic gradients across the sarcolemma and help preserve action potential even as fatigue develops.

### Limitations

Some limitations need to be taken into account before generalizing the presented results. First, the study has a relatively small sample szie (11 endurance-trained and 11 recreationally active males without endurance training background), which may reduced statitical power to detect beween-group difference, increased the probability of a type II error and contributed to variability in responses. However, other investigations have used a simlar number of participants to assess the fatigability in trained and untrained populations (Mileva, Morgan & Bowtell, 2009; Callewaert et al., 2013). Secondly, the study only involoved young males, so the ability to generalize the results to females is limited. Future work should include female partricipants and examine potential effect of fatigue on sex and training background. Thirdly, the fatiguing protocol consisted of only a sustained submaximal isometric knee extension task. As isometric and dynamic fatigue may result in different neuophysiological strategies, future research should examine whether these group-specific differences are still evident during dynamic exercise. Finally, we acknowledge that MFCV is an indirect estimate and represents the global average of the active motor unit pool rather than the discrete properties of indivdiuasl muscle fibres. Future studies utilizing HDsEMG decompositon or intramuscular recordings could provide deeper insights into the recrutiment of specific motor unit populations.

### Practical applications

The current investigation provides insights into neuromuscular responses to a sustained isometric fatiguing exercise in individuals with and without endurance-trained background. This information could potentially be used in designing more effective personalised training programs that cater to the specific physiological characteristics of each group, thereby optimising performance enhancement while effectively managing fatigue, recovery and injury risk.

## Conclusions

In this study, no differences were observed in TTFs or post-fatigue MVIC% declines between ET and RA groups. Only the RA group showed significant reductions in EMG MDF and MFCV during the sustained fatiguing contraction, whereas they remained unchanged in ET group. Additionally, the MFCV-torque relationship was steeper, and MFCV at 60% MVIC plateau was greater in RA than in ET participants. Overall, these findings suggest that, while submaximal isometric fatiguing task induced similar levels of muscle fatigue in both groups, the underlying neuromuscular fatigue mechanisms differ, likely due to a higher proportion of type II fires and/or a greater relative area of type II fibres in RA group.

##  Supplemental Information

10.7717/peerj.20979/supp-1 Supplemental Information 1 Raw data

## Additional Information and Declarations

### Data Availability

The following information was supplied regarding data availability: The raw data are available in the Supplementary File.
